# Genomic heritability estimation for the early life-history transition related to propensity to migrate in wild rainbow and steelhead trout populations

**DOI:** 10.1002/ece3.1038

**Published:** 2014-03-20

**Authors:** Guo Hu, Chunkao Wang, Yang Da

**Affiliations:** 1 Heilongjiang River Fishery Research Institute, Chinese Academy of Fishery SciencesHarbin, 150070, China; 2 Department of Animal Science, University of MinnesotaSaint Paul, Minnesota, 55108

**Keywords:** Genomic heritability estimation, genomic prediction, *Oncorhynchus mykiss*, smoltification

## Abstract

A previous genomewide association study (GWAS) identified SNP markers associated with propensity to migrate of rainbow and steelhead trout (*Oncorhynchus mykiss*) in a connected population with free access to the ocean in Upper Yakima River (UYR) and a population in Upper Mann Creek (UMC) that has been sequestered from its access to the ocean for more than 50 years. Applying genomic heritability estimation using the same dataset, we found that smoltification in the UYR population were almost completely determined by additive effects, with 95.5% additive heritability and 4.5% dominance heritability, whereas smoltification in the UMC population had substantial dominance effects, with 0% additive heritability and 39.3% dominance heritability. Dominance test detected one SNP marker (R30393) with significant dominance effect on smoltification (*P* = 1.98 × 10^−7^). Genomic-predicted additive effects completely separated migratory and nonmigratory fish in the UYR population, whereas genomic-predicted dominance effects achieved such complete separation in the UMC population. The UMC population had higher genomic additive and dominance correlations than the UYR population, and fish between these two populations had the least genomic correlations. These results suggested that blocking the free access to the ocean may have reduced genetic diversity and increased genomic similarity associated with the early life-history transition related to propensity to migrate.

## Introduction

Anadromy is a complex type of life cycle history found among several fish species including lampreys, sturgeons, basses, and salmonids (Dingle 1991; Stefansson et al. 2008). *Oncorhynchus mykiss* is a salmonid species, which exhibit tremendous life-history variation, and is a very interesting study object for the research of migration-related traits (Hoar 1976, 1988; Stefansson et al. 2008). Usually, the anadromous type of *O. mykiss* is called steelhead trout, and the nonanadromous residents are called rainbow trout. Steelhead and rainbow trout occur in sympatry throughout the species range in rivers and lakes with access to the sea (Behnke 2002), and either type of *O. mykiss* could be derived from one another (Zimmerman and Reeves 2000; Pascual et al. 2002; Thrower et al. 2004a). After a period of juvenile growth, steelhead trout reared in freshwater will undergo a complex early life-history transformation related to the propensity to migrate (smoltification) to the sea. Smoltification is a unique feature of salmonid anadromy and involves a number of developmental changes in the biochemistry, physiology, morphology, and behavior of the juvenile salmonid (Hoar 1976, 1988; Dellefors and Faremo 1988; Dickhoff et al. 1997; Behnke 2002; Stefansson et al. 2008). This early life-history transition change is also termed as “parr-smolt” transformation for juvenile salmonid (Hoar 1976).

Propensity to migrate in wild rainbow and steelhead trout population had heritable genetic component, and several quantitative trait loci (QTL) were identified (Nichols et al. 2008). Other studies suggested high gene flow through interbreeding of the anadromous and nonanadromous type of *O. mykiss* (Docker and Heath 2003; Narum et al. 2004; Olsen et al. 2006; Araki et al. 2007). Recently, a genomewide association study (GWAS) reported 504 single-nucleotide polymorphism (SNP) markers associated with the propensity to migrate using two populations with and without access to the ocean (Hecht et al. 2013). The “parr-smolt” transformation was reported to have a heritability of 0.726 estimated using pedigree information of a hybrid population of *O. mykiss* between the anadromous and derived freshwater fish (Thrower et al. 2004b). However, heritability of the early life-history transition directly estimated from the wild populations of the rainbow and steelhead trout remains unknown. Traditional methods for estimating heritability require the knowledge of pedigree relationships that are unavailable for wild fish populations. With the availability of genomewide SNP markers, genetic relationships among individuals without pedigree information can be estimated (VanRaden 2008; Hayes et al. 2009; Hayes and Goddard 2010; Goddard et al. 2011; Yang et al. 2011) and additive and dominance heritability can be estimated without requiring knowledge of pedigree relationships (Da and Wang 2013; Da et al. 2014). Genomic heritability estimates of additive and dominance effects will provide an understanding of the whole-genome contribution to a phenotype.

In this study, we assess the total genomic contribution of additive and dominance effects to the early life-history transition related to propensity to migrate, to study the patterns of genomic predictions of migratory and nonmigratory fish, to estimate genomic relationships among wild fish, and to assess the type of genetic effects of SNP markers associated with propensity to migrate using a publically available GWAS data of Hecht et al. (2013).

## Materials and Methods

### Samples of rainbow and steelhead trout

The GWAS data of Hecht et al. (2013) have two samples. The sample of Upper Yakima River population (UYR) with free access to the ocean had 127 fish and was collected from the Upper Yakima River in the state of Washington, USA. The sample of Upper Mann Creek population (UMC) with 55 fish was from the Upper Mann Creek, a tributary of the Snake River in Idaho, USA, which has been sequestered from its access to the ocean for more than 50 years by a hydropower dam since 1958 (Holecek et al. 2012). The early life-history transition related to propensity to migrate was defined as a binary trait “SMOLT”, with “1” indicating fish propensity to migratory and “2” indicating fish propensity to nonmigratory. Of the 127 fish in the UYR population, 29 were migratory, 98 were nonmigratory, and seven of the 29 migratory fish had missing sex information. Of the 55 fish in the UMC population, 28 were migratory, 27 were nonmigratory, and four migratory fish had missing sex information (Hecht et al. 2013). A total of 11,196 SNPs were genotyped for the two samples. SNP loci with missing genotypes exceeding 20% of all individuals were removed, and 8,442 loci satisfied this requirement. We further required 5% minor allele frequency (MAF) in the combined samples, and the number of SNP markers was reduced to 5,215 for genomic estimation of variance components and genomic prediction.

### Genomic heritability, predictions, and additive and dominance relationships

#### Statistical model

Genomic estimates of additive and dominance heritabilities of SMOLT in each sample were obtained using the mixed model based on the quantitative genetics model that partitions a genotypic value into breeding value and dominance deviation (Da and Wang 2013; Da et al. 2014). The mixed model can be written as follows: 

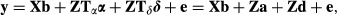
1

where **b** = fixed sex effects as in the GWAS model for the same data (Hecht et al. 2013), **a** = **T**_*α*_***α*** = genomic breeding values and **d** = **T**_*δ*_***δ*** = genomic dominance deviations, ***α*** = gene substitution effects, and ***δ*** = dominance effects. The variance–covariance matrices are as follows: var(**a**) = 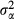 **A**_g_ = 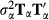, var(**d**) = 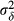 **D**_g_ = 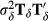, and 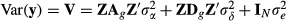. Let 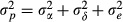 = phenotype variance, where 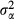 = additive variance, 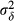 = dominance variance, and 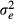 = residual variance. **A**_g_ is the additive genomic relationship matrix and **D**_g_ the dominance genomic relationship matrix. Then, additive heritability (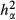), dominance heritability (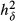) and heritability in the broad sense (*H*^2^) were calculated as follows: 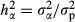, 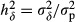, and 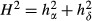. Genomic estimates of additive and dominance heritabilities of SMOLT, genomic prediction of additive effects, dominance effects and total genetic values, and genomic additive and dominance relationships were carried out by using GVCBLUP (Wang et al. 2013). The GCTA software (Yang et al. 2011) was used to estimate additive heritability as a confirmation for GVCBLUP. The GCTA does not have a feature to analyze dominance. Under the case–control model assumptions, the link function for binary data was the probit link. The relationship between the phenotypic observations and the liabilities on the unobserved continuous scale could be expressed by the following probit transformation (Dempster and Lerner 1950; Gianola and Foulley 1983; Kadarmideen et al. 2000; Lee et al. 2011, 2012):



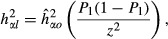
2

where 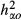 is the heritability of the observed scale; 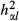 is the heritability of the liability scale linked to the observed scale; *z* is the height of the standard normal probability density function at the truncation threshold; *P*_*1*_ is the true population prevalence for the trait.

Genomic predictions of additive effects, dominance effects, and the total genotypic values as the summation of additive and dominance effects were calculated at the last iteration of the GREML estimation. Additive and dominance relationships were calculated using Definition III implemented by the GCORRMX program in GVCBLUP (Wang et al. 2013). The multiple tests for differences in means of genomic additive and dominance correlations between UYR and UMC populations were carried out using the R package (R Core Team 2012).

### Test of additive and dominance effects of genomewide SNP markers

Due to the wild population in the same environment for 50 years and had no pedigree information, a simple general linear model was used to deal with the data, and the model can be written as follows:



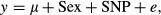
3

where *y* is the dependent variable (phenotypic observations), *μ* is population mean, *sex* effect as the only fixed effect in the model according to the study described by Hecht et al. (2013), and *e* is the random error. The significance of three effects, including total marker effect, additive effect, and dominance effect, are tested for each SNP at same time. The significance threshold is adjusted by a Bonferroni correction (5215 independent tests using the same dataset by a significant threshold being determined as *P* < 0.05), and the threshold of significance test was finally determined as *P *<* *3.2 × 10^−6^. Tests of additive and dominance effects of genomewide SNP markers were carried out by using the least squared test implemented by the EPISNP program (Ma et al. 2008).

## Results

### Genomic heritability estimates using genomewide SNP markers

For the SMOLT phenotypic values on the original observed scale using the mixed model with additive effect only by deleting dominance effect from Equation (1), both GVCBLUP and GCTA had genomic-additive heritability estimates of 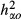 = 1.00 in the UYR population and 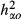 = 0.00 in the UMC population. The liability model adjustment to the observed scale using Equation (2) resulted in a heritability estimate of 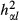 = 1.93 for UYR population. This was not surprising because heritability estimate on the liability scale exceeds “1” if the heritability estimate on the original scale exceeds 0.64 (2/*π*) (Lynch and Walsh 1998). Due to this known problem of heritability estimates on the liability scale, we use the heritability estimates on the original scale in discussion to follow.

For the mixed model of Equation (1) with both additive and dominance effects, the UYR population had high additive heritability and the UMC population had no additive heritability, whereas the UYR population had little dominance heritability and the UMC population had substantial dominance heritability (Table 1). In the UYR population, 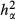 = 0.955, 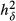 = 0.045, and *H*^2^ = 1. In the UMC population, 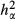 = 0, 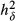 = *H*^2^ = 0.393. These results indicated that SMOLT in the UMC population had substantial dominance effects but additive or allelic effects were either lost or inactive possibly due to the 50 years of dam blocking to fish migration. In contrast, SMOLT in the UYR population with free access to the ocean was nearly completely affected by additive effects.

**Table 1:** GREML estimates of variance components of additive and dominance effects for the SMOLT trait in the Upper Yakima River population (UYR) and Upper Mann Creek population (UMC) using genomewide SNP markers.

Population	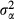	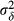	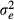	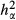	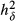	*H* ^2^
UYR	0.094	0.0044	2.6 × 10^−28^	0.955	0.045	1.000
UMC	8.3 × 10^−59^	0.097	0.15	0.000	0.393	0.393

### Patterns of genomic prediction

**Figure 1:**
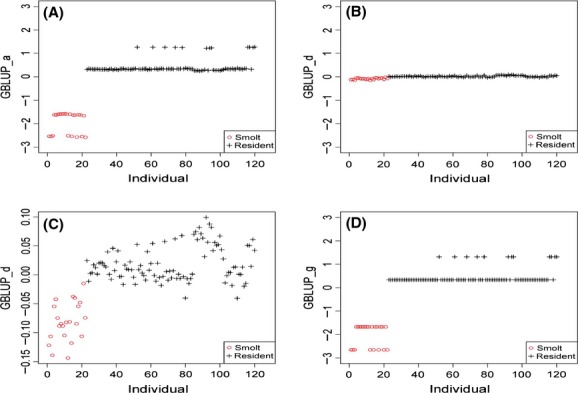
Patterns of genomic prediction for smoltification in Upper Yakima River population. (A) Genomic-predicted additive effects (GBLUP_a) of all individuals showed that Smolt fish had GBLUP_a > 0 and Resident fish had GBLUP_a < 0. Within Smolt or Resident, two groups of fish had distinct GBLUP_a values, indicating genome stratification of Upper Yakima River population into two subpopulations. (B) Genomic-predicted dominance effects (GBLUP_d) were nearly “0” for all individuals. (C) Enlarged GBLUP_d values separated most Smolt fish from Resident fish but failed to distinguish some fish. (D) Genomic-predicted genetic values (GBLUP_g) values had the clearest separation of Smolt fish from Resident fish. Within Smolt or Resident, two groups of fish had distinct GBLUP_g values with less variation than GBLUP_a values in A).

**Figure 2:**
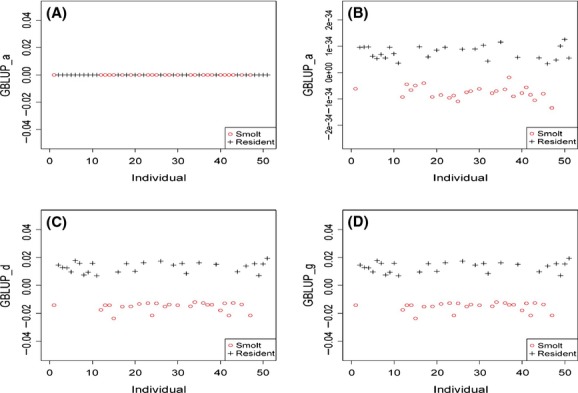
Patterns of genomic prediction for smoltification in blocked population. (A) Genomic-predicted additive effects (GBLUP_a) of all individuals were nearly “0” for all individuals. (B) Enlarged GBLUP_a values surprisingly separated all Smolt fish from Resident fish, although the original GBLUP_a values were nearly zero. (C) Genomic-predicted dominance effects (GBLUP_d) separated Smolt fish from Resident fish. (D) Genomic-predicted genetic values (GBLUP_g) had virtually identical patterns as GBLUP_d.

Genomic prediction of SMOLT had patterns in parallel to the estimated genomic contributions to SMOLT. In the UYR population, additive genomic prediction (GBLUP_a) completely separated migrate and nonmigrate fish (Fig. 1A), and dominance genomic prediction (GBLUP_d) was nearly zero for all individuals (Fig. 1B) but the enlarged GBLUP_d distinguished most migrate fish from nonmigrate fish (Fig. 1C), and genomic-predicted total genetic value (GBLUP_g = GBLUP_a + GBLUP_d) separated migrate and nonmigrate fish with least variations (Fig. 1D). The patterns of GBLUP_a and GBLUP_g showed that the UYR population had a genomic stratification with two nonoverlapping groups (Figs 1A and D). In the UMC population, GBLUP_a values for all individuals were virtually “0”, about 10^−59^ (Fig. 2A). However, enlarged GBLUP_a surprisingly separated migratory fish from nonmigratory fish completely, with GBLUP_a > 0 for migratory (Smolt) fish, and GBLUP_a < 0 for nonmigratory (Resident) fish (Fig. 2B). GBLUP_d and GBLUP_g distinguished migratory fish from nonmigratory fish completely (Fig. 2C and D). The patterns of genomic predictions discussed above were consistent with the results of genomic heritability estimates.

### Genomic additive and dominance correlations

Genomic correlations are useful measures of genomic similarity among individuals. The UMC population had higher genomic additive and dominance correlations than the UYR population, and fish between the UMC and UYR populations had the least genomic additive and dominance correlations (Table 2). The mean of genomic additive correlation in the UMC population was nearly five times the mean value in the UYR population, and the mean of genomic dominance correlation in the UMC population was about 1.5 times larger than the mean in the UYR population. Multiple significance tests for the means of UYR, UMC and interpopulation showed that genomic additive and dominance correlations in the UMC population were significantly higher than in the UYR population, and the average additive or dominance correlation in the UYR population or UMC population was significantly higher than the interpopulation correlations between fish in the UYR population with fish in the UMC population (*P *<* *0.05).

**Table 2:** Genomic additive and dominance correlations for the individuals within and between the Upper Yakima River population (UYR) and Upper Mann Creek population (UMC) of rainbow and steelhead trout.

Population	Genomic-additive correlation (Mean ± SE)	Genomic dominance correlation (Mean ± SE)
UYR	0.015 ± 0.00052	0.020 ± 0.00053
UMC	0.074 ± 0.0026	0.033 ± 0.00072
Interpopulations	−0.042 ± 0.00047	0.015 ± 0.00031

All pairwise comparisons were statistically significant with *P *<* *0.05.

### Significance test of additive and dominance effects of SNP markers

**Figure 3:**
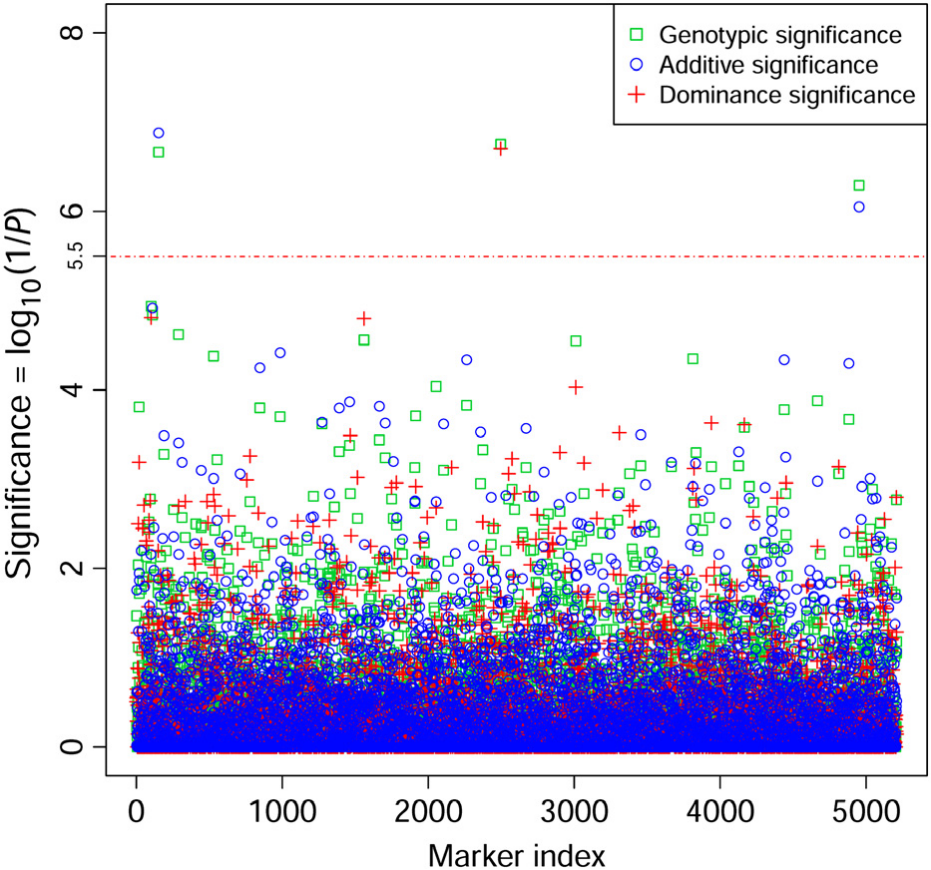
Statistical tests for genotypic, additive and dominance effects of SNP markers on SMOLT in Upper Yakima River population (UYR).

**Figure 4:**
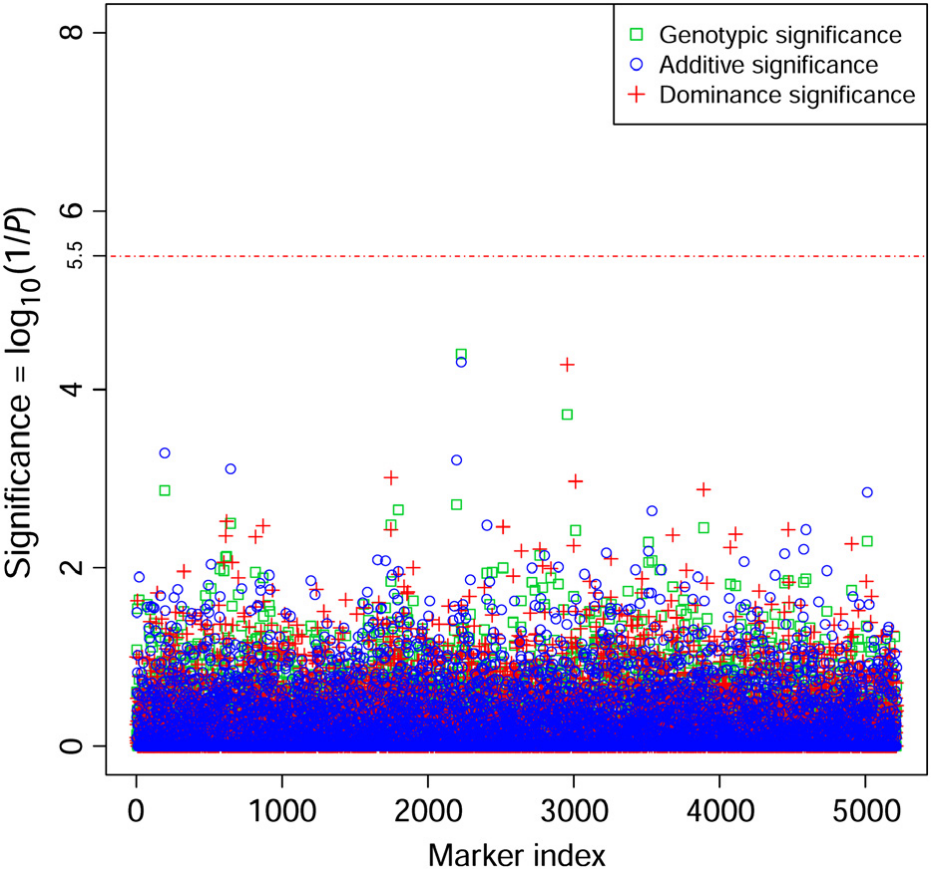
Statistical tests for genotypic, additive and dominance effects of SNP markers on SMOLT in Upper Mann Creek population (UMC).

The significance tests of additive and dominance SNP effects for SMOLT were conducted in the UYR population (Fig. 3) and in the UMC population (Fig. 4) separately. Three SNPs reached the 5% genomewide significance with the Bonferroni correction (*P *<* *3.2 × 10^−6^) in the UYR population, including R01916, R48563 and R30393 (Fig. 3, Table 2), but no genetic effect reached the 5% genomewide significance in UMC population (Fig. 4). The three significant SNP markers were among the four most significant SNP markers in the UYR population from the GWAS by Hecht et al. (2013), but we determined that R01916 and R48563 had highly significant additive effects and R30393 had highly significant dominance effect. The fourth significant SNP in Hecht et al. (R12248) had additive effect by our test but this effect did not reach the 5% genomewide significance (*P *=* *1.99 × 10^−4^). These test results were in agreement with the genomic heritability estimates in the sense that the significant marker effects were mostly additive effects but significant dominance effect also existed in the UYR population (Table 3).

**Table 3:** Statistical tests for genotypic, additive and dominance effects of four significant SNP markers for SMOLT in previously reported genomewide association analysis in the Upper Yakima River population (UYR).

SNP	Position	P_m	P_a	P_d
R30393	Unknown	1.78 × 10^−7^	0.0242	1.98 × 10^−7^
R01916	63.5 cM *Omy16*	2.19 × 10^−7^	1.32 × 10^−7^	0.0651
R12248	0.92 cM *Omy12*	1.99 × 10^−4^	3.80 × 10^−5^	0.961
R48563	Unknown	5.17 × 10^−7^	8.98 × 10^−7^	0.0152

“Position” is according to the RAD linkage maps in Miller et al. (2012); P_m = *P* value of marker genotypic effect, P_a = *P* value of additive effect, P_d = *P* value of dominance effect.

## Discussion

Smoltification is under complex genetic control, and uncovering the molecular mechanisms of migration will help to understand the life cycle history for *O. mykiss* (Nichols et al. 2008; Stefansson et al. 2008; Hecht et al. 2012; Miller et al. 2012). Our results showed a very high additive heritability (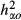 = 0.955) for SMOLT in the UYR population, and nonexistence of additive heritability (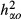 = 0.00) in the UMC population. Using pedigree information, Thrower et al. (2004b) estimated additive heritability of the transition of life cycle history (SMOLT) to be 0.762, which is between our estimate 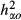 = 0.955 in the UYR population and 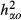 = 0.00 in the UMC population. Our results and the result of Thrower et al. should be consistent because the population used by Thrower et al. had crosses between lines of wild anadromous steelhead (similar to our UYR population) and wild resident (lake) rainbow trout originally derived from the same anadromous stock 70 years earlier (similar to our UMC population). Our results were mostly in agreement with those of their study. Compared with the estimate of Thrower et al. (2004b), the additive heritability for SMOLT from this study could be an overestimate. Different environmental conditions and sample sizes might be possible reasons for the differences between our results and the estimate of Thrower et al. (2004b). Other factors that could have contributed to the difference in the heritability estimates but could not be recorded include genetic differences and different genotype-by-environment interactions between the UYR–UMC populations in this study and the Sashin Lake population in the study of Thrower et al. (2004b). However, according to the methods of the sample collection described by Holecek et al. (2012) and Hecht et al. (2013), we tend to believe the samples in this study was random enough and could be representative of the true population. Thus, the genomic heritability estimates for the SMOLT should be informative and should contribute to the understanding of the genetic component of SMOLT. Potential reasons for the difference between the high and low heritabilities in the two populations include different history for the two populations (Hoffmann and Merila 1999). Selection could have played a role but the available data sets do not have information to make inference about the role of selection.

Results of genomic heritability estimates revealed large genetic difference associated with SMOLT between the UYR and UMC populations. SMOLT was completely explained by genetic factors in the UYR population and had substantial dominance effects in the UMC population. The three significant SNP markers in the UYR population from the GWAS in this study, and we determined that R01916 and R48563 had highly significant additive effects and R30393 had highly significant dominance effect. Almost all genetic effects were additive effects and dominance effects were only 4.5% in the UYR population, whereas dominance effects were substantial but additive or allelic effects were lost in the UMC population. It is unknown whether this loss was due to the loss or inactivation of alleles associated with SMOLT in the UMC population. However, evidence presented 40% of the genes in returning steelhead came from wild resident rainbow trout, rather than other steelhead trout (Araki et al. 2007), and wild rainbow trout was critical to health of steelhead populations. This implies that gene flow was blocked between the wild rainbow and steelhead trout populations and might have changed the genetic architecture of SMOLT. The results of genomic correlations imply another consequence of dam blocking: potentially increased genomic similarity and reduced genetic diversity in the UMC population. In addition, adaptive ecological differentiation for isolated populations was also promoted by adaptive local selection (Richter-Boix et al. 2013).

Genomic prediction of additive and dominance effects had several interesting results. First, genomic-predicted additive effects in the UYR population and genomic-predicted dominance effects in the UMC population were able to distinguish migratory fish from nonmigratory fish with 100% accuracy. This result was consistent with the results that additive effects were the primary genetic effects in the UYR population, and dominance effects were the primary genetic effects in the UMC population. Second, the enlarged additive genomic prediction values (GBLUP_a) in the UMC population perfectly recognized migratory and nonmigratory fish although the original GBLUP_a values were virtually zero, around 10^−59^. It is unknown whether this surprising result would hold in general or was due to chance. Third, patterns of genomic-predicted additive effects and genetic values in the UYR population revealed a clear population subdivision into two nonoverlapping groups.

In summary, evidence presented in this study indicated extremely high additive heritability in the anadromous population and extremely low additive heritability in the resident population for SMOLT. These results suggested that blocking free access to the ocean may have reduced genetic variation and increased genomic similarity associated with the early life-history transition related to propensity to migrate.

## Data Accessibility

The phenotypic and genotype datasets were publically available data from the genomewide association study of Hecht et al. (2013).
